# What radiolabeled FAPI pet can add in breast cancer? A systematic review from literature

**DOI:** 10.1007/s12149-023-01852-x

**Published:** 2023-06-21

**Authors:** Laura Evangelista, Luca Filippi, Orazio Schillaci

**Affiliations:** 1 Nuclear Medicine Unit, Department of Medicine (DIMED), University of Padua, Via Giustiniani, 35128 Padua, Italy; 2 Department of Nuclear Medicine, Santa Maria Goretti Hospital, Via Canova 3, 04100 Latina, Italy; 3 Department of Biomedicine and Prevention, University Tor Virgate, Rome, Italy

**Keywords:** 68 Ga-FAPI, Positron emission tomography–computed tomography, Breast neo-plasms, Fluorodeoxyglucose F18

## Abstract

To provide an overview of the current available data about FAPI PET in breast cancer patients, with a perspective point of view. A literature search for studies about FAPI PET in the last 5 years (from 2017 to January 2023) was carried out on MEDLINE databases, such as PubMed, EMBASE, Web of Science and Google Scholar using the following keywords: “PET” AND “FAPI” AND “Breast Cancer” AND “Fibroblast imaging”. The Critical Appraisal Skills Program (CASP) checklist for diagnostic test studies was used for testing the quality of selected papers. 13 articles were selected, including 172 patients affected by breast cancer who underwent FAPI-based PET images. CASP checklist was used in 5/13 papers, demonstrating a general low quality. Different types of FAPI-based tracers were used. No difference in terms of FAPI uptake was reported based on the histopathological characteristics, such as immunohistochemistry and grading of breast cancer. FAPI demonstrated more lesions and yielded much higher tumor-to-background ratios than 2-[18F]FDG. Preliminary experiences with FAPI PET in breast cancer showed some advantages than the current available 2-[18F]FDG, although prospective trials are needed to further evaluate its diagnostic utility in clinical practice.

**Supplementary Information:**

The online version contains supplementary material available at 10.1007/s12149-023-01852-x.

## Introduction

Breast cancer is the most frequent cancer and the second major cause of tumor death in women, after lung cancer [1]. It is defined as an heterogenous cancer due to various factors, such as tumor type, histological grade, lymph node metastasis, estrogen receptor (ER), progesterone receptor (PR), and human epidermal factor receptors 2 (HER2). All these features affect the treatment approach and the prognosis. Breast cancer biology represents a key factor for guiding the appropriate therapy; therefore, the identification of specific targets, often expressed on cancer cells, can be used for diagnosis and therapy, thus improving therapeutic outcome. Several tumor entities, such as breast, colon, and pancreatic carcinomas, are characterized by a strong desmoplastic reaction [2]. The presence of cancer-associated fibroblasts and extra-cellular fibrosis is associated with the gross tumor mass. Conversely from the normal fibroblasts, the cancer-related fibroblasts express a specific protein, named fibroblast activation protein (FAP). FAP is a transmembrane glycoprotein consisting of an extra-cellular and intra-cellular domain and a transmembrane component [3]. It represents one of the crucial components of the extra-cellular matrix and modulates or remodels the tumor microenvironment. For its peculiarity, fibroblast activating protein inhibitor (FAPI) has gained an important role as therapeutic target in a variety of human malignancies. Consequently, also FAPI-based radiopharmaceutical agents were produced, firstly with the aim of exploiting FAPI as a diagnostic target, but after as a potential theragnostic agent [4]. In the last years, different type of radiopharmaceuticals has been tested, either labeled with [68 Ga]Ga, but also with [177Lu]Lu or [225Ac]Ac.

Molecular imaging in breast cancer has a long research history, including whole-body PET, PET/CT and PET/MR scanning, and also dedicated systems either with SPECT or PET [5]. However, the clinical application of 2-[18F]FDG PET/CT is currently limited to the evaluation of locally or metastatic breast cancer, due to its limited sensitivity in detecting small breast lesions, micrometastases and some tumors with specific biological features (i.e., lobular carcinoma or low-grade breast tumors) [6, 7]. Many authors have recently demonstrated the potential clinical utility of FAPI-based PET in patients affected by breast cancer, trying to solve the current imaging gap. Herein, we provide an overview of the current available data about FAPI PET in breast cancer patients, with a perspective point of view.

## Materials and methods

### Literature search

Two authors performed the literature search, study inclusion, and data extraction. A literature search for studies about FAPI PET in the last 5 years (from 2017 to January 2023) was carried out on MEDLINE databases, such as PubMed, EMBASE, Web of Science and Google Scholar using the following keywords: “PET” AND “FAPI” AND “Breast Cancer” AND “Fibroblast imaging”. No limits were applied to the search strategy. Congress materials, reviews, letters to editors, editorials and clinical cases were excluded. After the recovery of the PDF files, the references of the studies already selected were checked.

From each study, the following data were recovered: type of the study (prospective, retrospective, etc.), year and geographical origin, sample size, setting of disease, type of FAPI agent, and comparative data with other radiopharmaceuticals.

### Quality of the selected studies

Selected imaging studies were analyzed using a modified version of the Critical Appraisal Skills Program (CASP) (https://casp-uk.net/aboutus, accessed on 1st February 2023) checklist for diagnostic test studies. Critical appraisal was performed by 2 reviewers, and discrepancies, if any, were resolved by discussion among researchers.

## Results

**Fig. 1:**
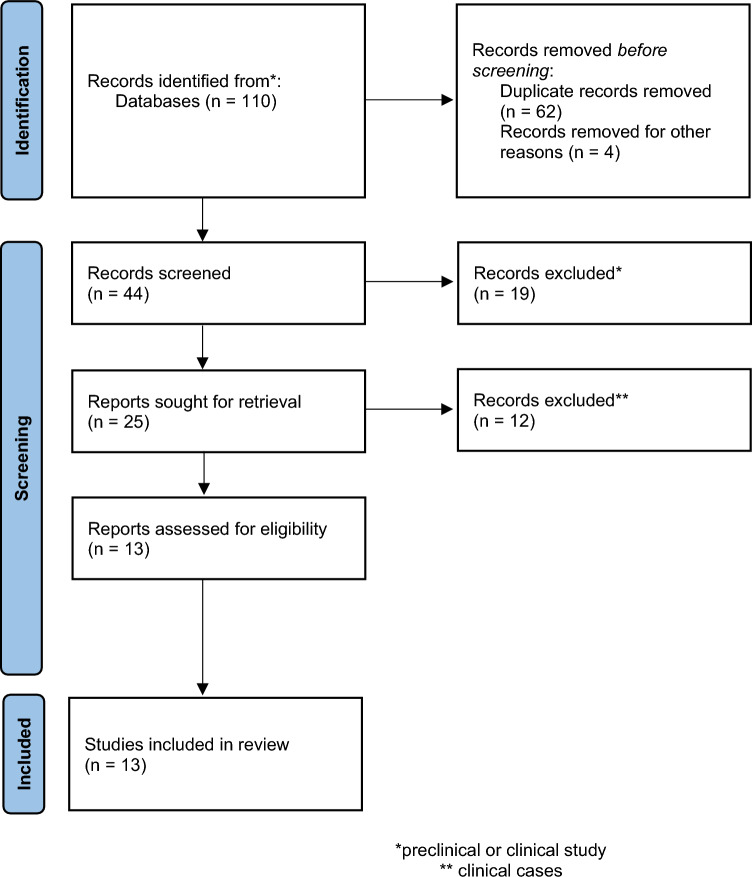
PRISMA flow-chart of the identified and selected papers

Based on the search criteria, 13 articles were selected (Fig. 1). In Table 1 are reported the main characteristics of the included reports. Totally, 172 patients affected by breast cancer underwent FAPI-based PET images. In most selected papers (*n* = 8; 62%, [8–15], FAPI-based PET/CT was used in other oncological diseases further than breast cancer. PET/MR was employed only in 2 studies [16, 17]. Different types of FAPI-based tracers were used; [Ga-]Ga-FAPI-04 was more often used [8–10, 12, 15, 18–20]. The setting of disease was not always available or not clearly stated, although restaging phase was more often a criterion of selection. The quality of papers was tested in only those considering patients affected by breast cancer (*n* = 5) [16–20]. Based on the CASP analysis, the quality of papers was generally low, because the standard of reference was often missed, too limited patient population were enrolled and only a descriptive analysis was made (Table 2 and Supplementary Table 1). However, the limited quality of the currently available papers represent an important challenges for the future clinical trials or studies.

**Table 1:** Characteristics of the included studies

Authors, referenes	Country	Type of study	Year of Pub	Study population	N pts	Setting of disease	Comparison with 2-[18F]FDG	Type of tracer (Injected dose)
Lindner et al. [8]	Germany	P	2018	Mixed	2	Restaging	No	[68 Ga]Ga-FAPI-04 (80 nmol/GBq)
Kratochwil et al. [9]	Germany	R	2019	Mixed	12	Not clear	No	[68 Ga]Ga-FAPI-04 (122–312 MBq)
Ballal et al. [11]	India	P	2020	Mixed	19	Staging and restaging	Yes	[68 Ga]Ga- DOTA.SA.FAPi (mean: 174 MBq)
Chen et al. [10]	China	P	2020	Mixed	4	Staging and restaging	Yes	[68 Ga]Ga-FAPI-04 (180–220 MBq)
Komek et al. [18]	Turkey	P	2021	Breast cancer	20	Staging and restaging	Yes	[68 Ga]Ga-FAPI-04 (2 MBq/Kg)
Dendl et al. [12]	Germany	R	2021	Mixed	14	Not clear	Yes	[68 Ga]Ga-FAPI-04 (52–325 MBq)
Elboga et al. [19]	Turkey	R	2021	Breast cancer	48	Staging and restaging	Yes	[68 Ga]Ga-FAPI-04 (2 MBq/Kg)
Backhaus et al. [13]	Germany	R	2022	Mixed	8	Staging and Restaging	No	[68 Ga]Ga-Onco-FAP (163.3 ± 50 MBq)
Backhaus et al. [16]	Germany	R	2022	Breast cancer	19	Staging and Restaging	No	[68 Ga]Ga-FAPI-46 (149 ± 48 MBq)
Mona et al. [14]	US	P	2022	Mixed	2	Not clear	No	[68 Ga]Ga-FAPI-46 (174–185 MBq)
Airo’ Farulla LS et al. [15]	Italy	R	2022	Mixed	2	Restaging	Yes	[68 Ga]Ga-FAPI-04 (not available)
Eshet et al. [20]	Israel	P	2022	Breast cancer	7	Not clear	No	[68 Ga]Ga-FAPI-04 (185 MBq)
Backhaus et al. [17]	Germany	R	2022	Breast cancer	13	Restaging (post-NAC)	No	[68 Ga]Ga-FAPI-46 (99 ± 33 MBq)

^*^Comparison [68 Ga]Ga-FAPI-46; *NAC* neoadjuvant chemotherapy; *R* = retrospective; *P* = prospective

**Table 2:** CASP evaluation for 5 selected articles including breast cancer and FAPI PET

Questions		[18]	[19]	[16]		[20]	[17]
Final agreement from 2 appraisers							
1	Was there a clear question for the study to address?	Yes	Yes		Yes	Yes	Yes
2	Was there a comparison with an appropriate reference standard?	No	No		Yes	No	Yes
3	Did all patients get the diagnostic test and reference standard?	Can’t tell	Can’t tell		Yes	Can’t tell	Yes
4	Could the results of the test have been influenced by the results of the reference standard?	Can’t tell	Can’t tell		No	Can’t tell	No
5	Is the disease status of the tested population clearly described?	Yes	Yes		Yes	Yes	Yes
6	Were the methods for performing the test described in sufficient detail?	Yes	Yes		Yes	Yes	Yes
7	What are the results?	Sensitivity and specificity	Descriptive analysis		Descriptive analysis	Descriptive analysis	Descriptive analysis
8	How sure are we about the results? Consequences and cost of alternatives performed?	Too preliminary	Too limited experience		Too limited experience	Too limited experience	Preliminary data
9	Can the results be applied to your patients/the population of interest?	Yes	Yes		Yes	Yes	Yes
10	Can the test be applied to your patient or population of interest?	Yes	Yes		Yes	Yes	Yes
11	Were all outcomes important to the individual or population considered?	Yes	Yes		Yes	Yes	Yes
12	What would be the impact of using this test on your patients/population?	The opportunity to have an alternative imaging	Interesting impact, further evaluations are need		Interesting impact, further evaluations are need	Interesting with an appropriate effect	Interesting impact, further evaluations are need

As illustrated in Table 1, different types of FAPI tracers have been tested across the studies, although in the those including only breast cancer, [68 Ga]Ga-FAPI-04 and [68 Ga]Ga-FAPI-46 were mostly used [16–20]. Indeed, Lindner et al. [6] found that diagnostic PET/CT scans performing after 10 min, 1 h and 3 h from the injection of [68 Ga]Ga-FAPI-04 in 2 patients with metastatic breast cancer, demonstrated a robust accumulation of tracer in the metastases, in contrast to the normal breast tissue where the tracer uptake was low. Moreover, due to its low retention, longer dwell times and no significant increase in background activity, [68 Ga]Ga-FAPI-04 is suitable also for the theragnostic purpose. In the study by Mona et al. [14], [68 Ga]Ga-FAPI-46 was tested also in 2 patients affected by breast cancer, showing a strong correlation with FAP expression in the cancer, thus rendering it a suitable radiopharmaceutical agent both for the diagnostic and therapeutic approach.

The biodistribution of FAPI in women can be affected by the hormonal status. The study from Dendl et al. [12] found that maximum standardized uptake value (SUVmax) was higher in premenopausal than post-menopausal patients in endometrium and breast tissue. Conversely, tracer uptake was similar between the two categories of patients in the ovaries. The uptake of FAPI in primary and metastatic breast cancer lesions has been reported in all the selected papers. In primary tumor, the SUVmax ranged between 2.6 and 17.0 [19]; similarly, high uptake values were reported in all metastatic sites (such as lymph nodes, lung, liver and bone). Interestingly, no difference in terms of FAPI uptake was reported based on the histopathological characteristics, such as immunohistochemistry and grading [12, 18, 19]. However, Elboga et al. [19] noticed that in HER2 patients, the FAPI uptake was higher as compared to the other luminal subtypes. Furthermore, a slightly higher mean uptake was observed in BRCA 1/2-positive patients than negative patients with regards to all lesions [12].

As illustrated in Table 1, 6 papers were focused on the comparison between 2-[18F]FDG and FAPI [10–12, 15, 18, 19]. Totally, data for 107 patients were now available. As emerged by the studies, FAPI demonstrated more lesions and yielded much higher tumor-to-background ratios than 2-[18F]FDG. As illustrated in Table 3, in all sites of disease, FAPI uptake was higher than 2-[18F]FDG also by a semiquantitative point of view, particularly when SUVmax was used. Furthermore, also the sensitivity for the breast cancer was higher in FAPI than 2-[18F]FDG being equal to 100% vs. 78.2%, respectively [18]; conversely, a slightly decrease in specificity was reported (95.8% vs. 100%, respectively for FAPI vs. 2-[18F]FDG; [18]). However, based on the study from Kömek et al. [18], FAPI PET was able to detect more lymph nodes, and sub centimetric lesions in hepatic tissue. Indeed, in their study, the authors reported that the median size of only FAPI positive lesions was 9 mm, therefore under the standard 10 mm. Interesting comparative data were reported by Elboga et al. [19], indeed the authors found that an early treatment response with 2-[18F]FDG PET/CT can display false-negative results, while FAPI can detect lesions even within the first month of post-chemotherapy period. Patients considered as responders or with a stable disease after chemotherapy at 2-[18F]FDG PET/CT, were later reclassified as progressive after FAPI PET imaging, thus approaching to a correct clinical management. The ability of FAPI PET to detect small lesions after chemotherapy was reported also by Backhaus et al. [17]. The authors found that FAPI PET/MR was able to classify responders vs. non-responders to neoadjuvant chemotherapy, otherwise incorrectly evaluated by MR alone. The same group confirmed that the combination of FAPI with PET/MR can significantly improve the detection of primary breast tumors and regional lymph node disease than MR alone.

**Table 3:** Mean ± standard deviation or median (range) of the semiquantitative data from 2-[18F]FDG and FAPI in primary and in metastatic site of disease

Authors, references	Primary		Lymph node		Distant	
	FAPI	2-[18F]FDG	FAPI	2-[18F]FDG	FAPI	2-[18F]FDG
Ballal et al. [11]	6.5 ± 3.3* 4.9 ± 2.5**	6.2 ± 1.6* 4.7 ± 2.3**	– –	– –	– –	– –
Komek et al. [18]	17.4 (10.4–22.8)Ϯ	5.8 (1.3–15.5)Ϯ	16.7 (3.1–23.5)Ϯ	5.1 (1–12)Ϯ	9.2 (5.5–19)Ϯ (LiM) 6.1 (1.3–16.6)Ϯ (LM) 6 (3.7–15.1)Ϯ (BM)	6.1 (3.5–14)Ϯ (LiM) 2.6 (1.2–10.1)Ϯ (LM) 4.4 (2.5–8.1)Ϯ (BM)
Elboga et al. [19]	10 (2.6–17.00)Ϯ	3.1 (1.1–13.2)Ϯ	12.7 (4.1–36.6)Ϯ	3.9 (1.7–20.6)Ϯ	22.3 (20–23)Ϯ (LM) 17.9 (7.7–28)Ϯ LiM 14.9 (9.9–25)Ϯ BM	7.1 (2.2–14)Ϯ (LM) 3.5 (3.1–3.8)Ϯ LiM 3.2 (1.4–5.2)Ϯ BM

^*^SULpeak, **SULavg; ϮSUVmax; *LM* lung metastases; *LiV* liver metastases; *BM* bone metastases

## Discussion

Until to date, 172 patients affected by breast cancer were studied with radiolabeled FAPI PET/CT. Therefore, limited data are available for drawing final comments, particularly in an oncological disease with a high incidence and prevalence in females. However, many concepts can be extrapolated from the 13 available papers that evaluated the role of different FAPI agents, also in comparison with 2-[18F]FDG. Indeed, currently the glucose-based agent, 2-[18F]FDG, is still considered the most common PET agent in breast cancer imaging, but it is linked by many clinical issues that are currently unsolved. First, 2-[18F]FDG uptake is low in some histopathological types, i.e., luminal A (positive estrogen receptor and a low proliferation index), lobular cancer, and in HER2-positive disease, thus significantly affected its diagnostic performance in these settings. Recently, [18F]Fluoroestradiol ([18F]FES) has been introduced in the clinical practice for overpassing some limitations of 2-[18F]FDG in patients with lobular cancer and luminal A subtypes, but its availability is still limited at few centers. Second, 2-[18F]FDG cannot be used to distinguish between malignant and benign disease because of a low target/background ratio in small lesions and to the partial volume effect [25, 26], although the current availability of dedicated breast scanners. Third, current International guidelines suggested to use [18F]FDG as on optional imaging in stage II or stage III breast cancer, when conventional imaging is negative or inconclusive. The limited employment of this imaging technique in the initial staging of disease, also in case of locally advanced breast cancer, is relative to its limited sensitivity and specificity, although the rate of metastases in this setting can arrive to 40% [27]. Fourth, 2-[18F]FDG is not indicated for the evaluation of response to therapy, either in adjuvant and in neoadjuvant setting, depending by its limited ability to identify small residual cancer tissue. Finally, there is not a specific radiopharmaceutical agent that can be used either in diagnostic or in therapeutic field for breast cancer. Therefore, on the basis of the abovementioned limitations, some additional agents for PET imaging, and not only, are strongly required.

The expression of FAP on activated fibroblasts in tumor stroma was quantified in 1990, when also a high correlation was reported in breast cancer [21]. The decision for the best peptides for breast cancer in a large amount of available FAPI agents depends on a lot of considerations: (1) the ability to detect the cancer lesions, (2) a high tumor-to-background ratio, (3) the capacity of distinguish between benign and malignant breast lesions and (4) the opportunity to use it either for diagnostic or therapeutic purpose. Based on this assumption, in the present systematic review, emerged that [68 Ga]Ga-FAPI-04 showed a high SUVs in many studies and when compared to the others, such as [68 Ga]Ga-DOTA-SA-FAPI [9]. However, a proof of principle work on [68 Ga]Ga-DOTA-SA-FAPI PET/CT-guided radioligand therapy with 177Lu-DOTA-SA-FAPI exists, thus opening the way for its theragnostic application in breast cancer [22].

Although 2-[18F]FDG PET/CT is commonly used in recurrence, as previously mentioned, it is not generally recommended in initial staging of breast cancer [20]. Only aggressive invasive ductal breast cancer, such as triple negative and grade III tumors showed a moderate-high 2-[18F]FDG uptake, while a controversial uptake has been reported in HER2-positive tumors [23, 24]. FAPI uptake is not correlated with histopathological, molecular feature and tumor grade, being equally increased in all types of breast cancer. This behavior can solve the limitation of 2-[18F]FDG, with a strong clinical importance, either in patients with lobular cancer or in those with HER2-positive, luminal A and luminal B disease. Currently, preliminary data by Eshet et al. [20] reported the ability of FAPI PET in detecting more lesions than CT in seven women with lobular cancer, in many distant organs, such as orbits, posterior mediastinum, internal mammary, retroperitoneum and pelvis. Due to the limited available data about the correlation among histology, molecular subtypes and FAPI uptake, prospective studies are mandatory. Although, the independence of FAPI uptake from the biological characteristics of breast cancer can be an advantage in terms of lesions’ detection, FAPI PET cannot predict the aggressiveness of the lesion and therefore it cannot be considered a prognostic parameter. Probably, the combination of FAPI and 2-[18F]FDG would be suggested to cover both the information (either diagnostic or prognostic) in breast cancer patients. In this way, no current data about the correlation between prognosis and FAPI are available.

Some studies have demonstrated that an early treatment response with 2-[18F]FDG PET/CT can display false-negative results [28, 29]. To monitor the response to therapy, mainly in neoadjuvant setting is an important clinical issue for 2 main reasons: (1) the opportunity to early test the chemosensitivity of the tumor and (2) to early change the therapeutic scheme in case of treatment failure. To date, MR is the imaging of choice for these endpoints, but some studies have demonstrated its limited sensitivity and specificity also when compared to 2-[18F]FDG PET/CT [30]. The study by Elboga et al. [19] tried to overpass this limitation, using FAPI. The opportunity to recognize the presence of residual active disease, within one month from the start of chemotherapy, can be useful for planning the therapeutic management. Therefore, FAPI-based PET can help in this way and further evaluation should be performed, either in neoadjuvant, adjuvant or metastatic setting.

The advantages from FAPI in comparison to 2-[18F]FDG are relative also to the radiation burden and some practical issues. First, based on Table 1, the amount of injected radiolabeled FAPI was about 200 MBq (5.4 mCi), therefore with an effective whole-body dose of 1.56 ± 0.26 mSv for a PET scan. When including a low-dose CT scan (3.7 mSv), the dose is approximately 5.3 mSv in total [31]. Conversely, in the current clinical setting, the radiation burden of FDG in oncological setting range between 5 and 15 mSv for a PET/CT scan [32]. Furthermore, the opportunity to use digital or whole-body PET scanner can further reduce the radiation exposure in this setting of disease, that often affects young women.

Second, FAPI uptake is independent from the fasting and resting time; moreover the images can be obtained soon after 10 min from the tracer’s injection, because of its fast clearance and lower-off target accumulation. However, also some disadvantages have been reported from the use of FAPI in breast cancer as compared to 2-[18F]FDG. The first one is the influence on FAPI uptake due to the changes in hormone status, thus altering the interpretation of the images in premenopausal women [3, 12]. The differences in pre- and post-menopausal patients are relative to a major uptake in the health breast tissue that can influence the tumor-to-background ratio, but larger studies in these two categories of patients are required for better understanding the hormonal effects of the FAPI uptake. The second disadvantage of FAPI is the detection of more false-positive findings than 2-[18F]FDG due to various fibrotic processes, such as myelofibrosis, granulomatous disease, liver cirrhosis [19] and inflammatory processes (i.e., tuberculosis, [33]). These false-positive findings can reduce the specificity and the positive predictive value of FAPI PET, mainly soon after surgery, radiation therapy and during follow-up.

As largely known, MRI is better than CT in the evaluation of primary breast cancer, due to its high contrast resolution and also for the ability to differentiate between malignant and benign lesions in contrast-enhanced sequences. However, FAPI can be complementary to MRI, overpassing its limited specificity. Indeed, in the study by Backhaus et al. [16], no FAPI uptake was reported in patients with papilloma, ductal carcinoma in situ and in BIRADS-2 lesions. In another study by the same group [17], FAPI PET/MR was able to correctly identify all patients with a complete pathological response after neoadjuvant chemotherapy, otherwise missed by MRI alone. Although these encouraging preliminary results, additional data are needed, also considering the limited number of hybrid PET/MR scanner worldwide. Alternatively, combined PET and MR images would be considering in the future studies for improving the differential diagnosis in indeterminate breast lesions.

The present review form the literature has some limitations. First, the limited number of included papers. Second, several types of FAPI tracers were reported, but currently no specific indications on how can be better use in each disease has been extensively demonstrated. Third, the diagnostic performance of FAPI has not been reported in all papers, but only in the study by Kömek et al. [18]. Finally, some papers [13, 16, 17] were made by the same group of researchers, thus with a potential overlap of the patient population.

## Conclusions

In conclusion, preliminary experiences with FAPI PET in breast cancer are interesting for a lot of reasons: (1) the ability to detect more lesions than 2-[18F]FDG, (2) the independence of FAPI uptake from the molecular and histopathological features and (3) the opportunity to detect small lesions after chemotherapy, thus guiding to a further appropriate therapy. Future studies are warranted, in large population, to confirm these assumptions.

## Supplementary Information

Below is the link to the electronic supplementary material.

Supplementary file1 (PDF 114 KB)

## Data Availability

Not applicable.
