# Supplementary material for: What radiolabeled FAPI pet can add in breast cancer? A systematic review from literature
Source: Ann Nucl Med. 2023 Jun 21;37(8):442–50. doi: 10.1007/s12149-023-01852-x (PMC10345025; doi:10.1007/s12149-023-01852-x)
Supplement: Supplementary file 1 — Supplementary file1 (PDF 114 KB) [file 12149_2023_1852_MOESM1_ESM.pdf]

Supplementary Table 1. Description of the CASP checklist in 5 selected paper

		**[18]**	**[19]**	**[16]**	**[20]**	**[17]**
**N**	**Questions**					
1	Was there a clear question for the study to address?	P=breast cancer patients<br>I=FAPI PET/CT<br>C=FDG PET/CT<br>O=detection of primary and recurrent BC	P=breast cancer patients<br>I=FAPI PET/CT<br>C=FDG PET/CT<br>O=detection of LABC or metastatic disease	P=breast cancer patients<br>I=FAPI PET/MR<br>C=none<br>O=lymph node and distant staging	P=breast cancer patients<br>I=FAPI PET/CT<br>C=FDG PET/CT<br>O=detection of metastatic disease	P=breast cancer patients<br>I=FAPI PET/MR<br>C=MRI alone<br>O=identification of T and N status after NAC
2/3	Was there a comparison with an appropriate reference standard?/ Did all patients get the diagnostic test and reference standard?	MRI and histopathology	Not available	Histopathological assessment	Not available	Histopathological assessment
5	Is the disease status of the tested population clearly described?	Primary and recurrent BC	LABC or metastatic disease	Confirmed invasive breast cancer	Metastatic lobular breast cancer	Confirmed invasive breast cancer
7	What are the results?	1-diagnostic accuracy<br>2-detection rate<br>3-semiquantitative values	1-Semiqunatitative analysis<br>2-descriptive analysis	1-Detection rate for N and M<br>2-Tumor-to-background ratio for N	1-Detection rate for M	1- diagnostic performance<br>2- detection rate<br>3- tumor-to-background ratio for T and N
9	Can the results be applied to your patients/the population of interest?	The population of interest is made of women with primary or recurrent BC (all histological type) (age ranged between 32 and 65 yrs)	The population of interest is made of women with LABC or metastatic BC (all histological type) (mean age: 53 yrs)	The population of interest is made of women with invasive breast cancer (age ranged between 34 and 66 yrs)	The population of interest is made of women with metastatic lobular breast cancer (age ranged between 46 and 67 yrs)	The population of interest is made of women with invasive breast cancer (age ranged between 34 and 59 yrs)

10	Can the test be applied to your patient or population of interest?	FAPI PET/CT	FAPI PET/CT	FAPI PET/MR	FAPI PET/CT	FAPI PET/CT
11	Were all outcomes important to the individual or population considered?	Because FAPI PET/CT can help to detect more lesions than FDG PET/CT	Because FAPI PET/CT can help to detect more lesions than FDG PET/CT	Because FAPI PET/MR can help the detection of more nodal and distant metastases	Because FAPI PET/CT can detect metastatic lobular breast cancer otherwise missed by FDG PET	Because FAPI PET/MR can help the detection of residual disease after NAC
12	What would be the impact of using this test on your patients/population?	FAPI seems superior to FDG PET/CT (preliminary in 20 pts)	FAPI seems superior to FDG PET/CT	Being preliminary results, it is difficult to define the impact of FAPI PET/MR (also considering the limited availability of PET/MR scanners)	Being preliminary results, it is difficult to define the impact of FAPI PET/CT in this population	Being preliminary results, it is difficult to define the impact of FAPI PET/MR (also considering the limited availability of PET/MR scanners)

P=population; I=intervention; C=comparator; O=outcome; T=tumor, N=node; M=metastasis; NAC=neoadjuvant chemotherapy; BC=breast cancer; LABC=locally advanced breast cancer
